# Implementing Person-Centered, Clinical, and Research Navigation in Rare Cancers: The Canadian Cholangiocarcinoma Collaborative (C3)

**DOI:** 10.3390/curroncol32080436

**Published:** 2025-08-01

**Authors:** Samar Attieh, Leonard Angka, Christine Lafontaine, Cynthia Mitchell, Julie Carignan, Carolina Ilkow, Simon Turcotte, Rachel Goodwin, Rebecca C. Auer, Carmen G. Loiselle

**Affiliations:** 1 Department of Surgery, Faculty of Medicine, University of Ottawa, Ottawa, ON K1H 8M5, Canada; 2 Cancer Therapeutics Program, Ottawa Hospital Research Institute, Ottawa, ON K1H 8L6, Canada; 3 Patient Representative, Winnipeg, MB, Canada; 4 Patient Representative, Quebec City, QC, Canada; 5 Department of Biochemistry, Microbiology, and Immunology, Faculty of Medicine, University of Ottawa, Ottawa, ON K1H 8M5, Canada; 6 Département de Chirurgie, Service de Transplantation Hépatique et de Chirurgie Hépatopancréatobiliaire, Centre Hospitalier de l’Université de Montréal, Montreal, QC H2X 0C1, Canada; 7 Division of Medical Oncology, The Ottawa Hospital, University of Ottawa, Ottawa, ON K1H 8L6, Canada; 8 Gerald Bronfman Department of Oncology and Ingram School of Nursing, Faculty of Medicine and Health Sciences, McGill University, Montreal, QC H3A 2M7, Canada

**Keywords:** cancer navigation, biliary tract cancer, rare cancer, cholangiocarcinoma, hope, advocacy

## Abstract

Person-centered navigation (PCN) in healthcare refers to a proactive collaboration among professionals, researchers, patients, and their families to guide individuals toward timely access to screening, treatment, follow-up, and psychosocial support. PCN—which includes professional, peer, and virtual guidance, is particularly crucial for rare cancers, where affected individuals face uncertainty, limited support, financial strain, and difficulties accessing relevant information, testing, and other services. The Canadian Cholangiocarcinoma Collaborative (C3) prioritizes PCN implementation to address these challenges in the context of Biliary Tract Cancers (BTCs). C3 uses a virtual PCN model and staffs a “C3 Research Navigator” who provides clinical and research navigation such as personalized guidance and support, facilitating access to molecular testing, clinical trials, and case reviews through national multidisciplinary rounds. C3 also supports a national network of BTC experts, a patient research registry, and advocacy activities. C3’s implementation strategies include co-design, timely delivery of support, and optimal outcomes across its many initiatives. Future priorities include expanding the C3 network, enhancing user engagement, and further integrating its innovative approach into routine care.

## Simple Summary

Individuals diagnosed with biliary tract cancer often encounter significant challenges related to their rarity, including emotional distress, lack of information, limited support, and difficulties accessing timely treatment and research opportunities, including clinical trials. The Canadian Cholangiocarcinoma Collaborative (C3) brings together patients, caregivers, oncologists, researchers, and advocates from across Canada to address these issues. A dedicated clinical and research navigator provides one-on-one support to patients to help them access molecular testing, clinical trials, and a team of experts that can review their case. This navigation support is offered virtually for patients to access anywhere in Canada. Patients are also invited to take part in the C3 patient registry that collects real-world evidence to inform patient advocacy and drug approval submissions. The C3 represents a person-centered comprehensive initiative that can serve as a transferable model for other significant health conditions.

## 1. Introduction

Person-centered navigation (PCN) in healthcare involves proactive collaborations among professionals, patients, and their families to optimally guide them toward timely access to services and treatments [1]. PCN is particularly relevant to cancer care as it involves professional navigators (e.g., nurses), or lay navigators (e.g., trained volunteers or peers with lived experience), often using virtual means [2]. More recently, hybrid models have gained popularity by combining in-person and virtual PCN modalities [3]. The evidence gathered so far indicates that PCN addresses access barriers in a timely manner while increasing users’ satisfaction with care [4,5,6,7,8,9,10]. PCN research, however, has focused mainly on more prevalent cancer diagnoses (e.g., breast, prostate, and colorectal) with a notable gap regarding rare conditions such as Biliary Tract Cancers (BTCs) [11]. Although highly relevant, PCN implementation strategies remain underdeveloped and understudied in the context of BTC.

BTCs, which include cholangiocarcinoma and gallbladder cancers, are often asymptomatic in early stages and diagnosed late with a poor prognosis, with only ~15% of individuals experiencing progression-free survival at 12 months following standard chemotherapy [12,13,14]. The recent addition of checkpoint blockade immunotherapy to standard chemotherapy has improved the 12-month progression-free survival rates to 26.1%, offering some hope, but most succumb to the disease within two years. As such, individuals with BTC need to explore additional clinical research options to optimize treatment.

In Canada, the low BTC incidence (~800 new diagnoses/year), means that patients have limited access to specialists, clinical trials, and psychosocial support. In addition, costly targeted therapies add financial burden and distress. However, this group of patients often lacks psychosocial oncology support and has significant unmet cancer-related needs [15,16]. Therefore, implementing PCN early on in BTC care is a promising approach to address unmet needs, improve access, and enhance patient and caregiver experiences. The Canadian Cholangiocarcinoma Collaborative (C3) exemplifies the implementation of PCN in rare cancers through joint clinical and research navigation initiatives for maximum impact [17,18].

## 2. The Canadian Cholangiocarcinoma Collaborative (C3) Navigation Model

C3 was created in response to the pressing need for a national BTC network in Canada that is readily accessible by affected individuals. C3 brings together key stakeholders who have collaboratively developed the PCN program. As background, C3 has three core pillars: (1) C3-HOPE, which includes a national registry of individuals diagnosed with BTC, advocacy activities, support for patients and their families, and research opportunities; (2) C3-STAR, Canada’s first investigator-initiated clinical trial using tumor-infiltrating lymphocytes (TIL) therapy; and (3) C3-IMPACT, which focuses on establishing a Canadian BTC research pipeline. Across these pillars, C3 incorporates PCN and implementation strategies to optimize the design and delivery of its initiatives, starting with resource acquisition, then active implementation, and ultimately monitoring and sustainability.

C3 has an expanding network of patients, informal caregivers, clinicians, scientists, healthcare professionals, advocates, industry representatives, and government regulators. Each stakeholder plays a significant role. Patients and caregivers share firsthand experiences and needs; clinicians provide medical expertise and practical care guidance; scientists identify knowledge gaps and research priorities; advocates amplify patient voices; and industry partners offer resources and funding for drug development and clinical trials. Funded by the Canadian Cancer Society (CCS) and the Canadian Institutes of Health Research (CIHR), C3 welcomes over 150 BTC experts from across Canada, and at least 345 patients and their caregivers have reached out to date (data were extracted in June 2025). Referrals to C3 come from medical teams or directly through a user-friendly website (https://www.cholangio.ca/, accessed on 29 July 2025), social media platforms (Facebook, Instagram, X, and YouTube), and word-of-mouth from engaged patient partners and advocates. By integrating diverse perspectives, this collaboration ensures a comprehensive, effective, well-rounded approach while addressing gaps and priorities in BTC care.

**Figure 1:**
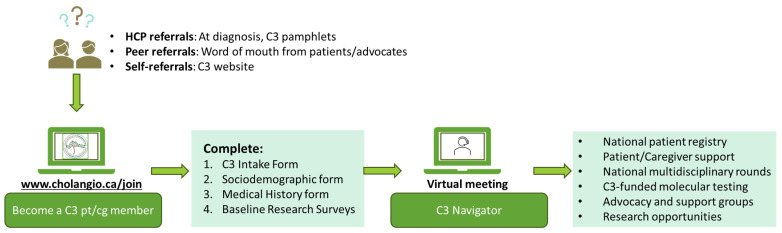
The C3 person-centered navigation (PCN) model.

C3 employs a virtual PCN model whereby the trained Navigator is the initial contact for patients and informal caregivers who join (Figure 1). The C3 “Research Navigator” term captures the unique role supporting patients at any stage of diagnosis and through complex clinical care and research opportunities. The navigator position mirrors a clinical research coordinator level of expertise with clinical and research experience. This navigator receives training through the C3 network, including professional coaching by the C3 founders and BTC oncologists. During the introductory informational session and discussion, the navigator learns more about patients’ medical history, provides personalized guidance, shares resources related to BTC, and highlights opportunities for involvement in clinical, psychosocial, and research activities. Conducted in English or French, these virtual sessions are accessible anywhere within Canada. The navigator also organizes informational sessions on clinical trials, including their importance, phases, types, enrollment, procedures, participant protections, advocacy, and navigating the Canadian healthcare system. In sum, these initiatives aim to empower patients to make informed decisions regarding their care and participation in research. C3 initiatives are described in Table 1.

### 2.1. National C3 Patient Registry

Patient registries are organized electronic databases that collect detailed information from individuals who have a specific disease or condition. Registries can help identify research questions, track equity trends, and provide deeper insights into the registry population through specific data points. For example, they can explore access to clinical trials and molecular testing and determine the frequency of a genetic mutation or the response rate to a targeted therapy. Canadian BTC registries exist but are limited to specific research ethics board-approved programs that may only collect data from a single center. This highlights the need for a national data registry to capture the BTC patient trajectory, inform regulatory decisions, attract drug developers, and link with existing national and international cancer registries.

The C3 Registry, approved by the Ottawa Health Science Network Research Ethics Board (OHSN-REB), enables the assessment of potential disparities in access to molecular testing, clinical trials, treatments, relevant clinical and patient-reported outcomes, and the generation of real-world evidence (RWE) regarding the efficacy and safety of emerging therapies. Designed to be “fit for purpose” the C3 Registry supports, for instance, the evaluation of a broad pipeline of emerging molecular therapies, including those under investigation in clinical trials and used off-label. For rare cancers, where large datasets are difficult to gather strictly through clinical trials, leveraging data and patient outcomes from investigational drugs accessed through an Exceptional Access Program or off-label use of drugs approved for other indications can be particularly valuable.

Canadian patients can enroll in the C3 Registry irrespective of their location or the cancer center treating them, ensuring equitable access. The C3 Registry includes individuals diagnosed with BTC (all forms of cholangiocarcinoma and gallbladder cancers), and (if applicable) their informal caregivers. Inclusion criteria for participating in C3 initiatives are as follows: a BTC diagnosis (any type, stage, or recurrence) or caring for someone with this diagnosis; 18 years or older; willingness and ability to complete surveys in English or French; having an email address, access to a remote device (e.g., iPad, laptop, smartphone), and an internet connection; and the capacity to provide consent for research purposes. Upon enrollment, patients sign an electronic agreement (i.e., terms and conditions reviewed and approved by the Ottawa Hospital Research Institute legal department) to share their information with C3, including medical records and molecular testing results, and/or agree that C3 may collect this information on their behalf. C3 facilitates the collection of relevant health record information—including clinic and inpatient notes, operative and procedural reports, laboratory results, radiology and pathology reports, and imaging—across all institutions where the patient is receiving care, acting on their behalf. Currently, records are procured and stored in the electronic medical records system, EPIC, at The Ottawa Hospital (TOH). A key principle of the C3 Registry is to allow patients (at their discretion) to store, access, and share their data with authorized individuals. The uniqueness of the C3 Registry lies in empowering patients to control their own data through the EPIC’s patient portal, MyChart^®^. Medical records are gathered and updated every six months to track the patient’s cancer history longitudinally. Relevant data fields are manually extracted by the C3 Registry Coordinator and populated into REDCap^®^ (Research Electronic Data Capture), a secure web-based application for managing research databases, with all collected data stored behind the TOH firewall. C3 collects supplementary information (approved by the ethics committee) through questionnaires sent to patients, capturing data not found in their medical records, such as family history, dietary habits, quality of life, levels of hope, and preferences for seeking cancer-related information.

In the future, we plan to transition our registry to the Citizen Health virtual platform, which is based on the same principle of empowering patients to have access to their own data, facilitating data collection, and providing them control of data sharing with providers and ongoing studies. The Citizen Health platform is already utilized by the International Cholangiocarcinoma Patient Registry in the United States, which includes >1000 individuals with cholangiocarcinoma worldwide.

### 2.2. National BTC Multidisciplinary Rounds

To build and maintain an effective research network, medical professionals must be informed about the latest clinical trials, targeted therapies, and available resources. Moreover, promoting connections among healthcare providers who care for BTC patients enhances knowledge sharing and encourages collaborative discussions about complex cases, including those that involve the interpretation of molecular reports. At the University Health Network (UHN), there is a highly effective molecular tumor board to help guide therapy for patients treated in those centers, but it is currently unavailable to patients across Canada. This is particularly beneficial for oncologists working in rural or smaller hospitals with limited experience treating BTC, particularly related to the interpretation of molecular reports and the use of molecularly targeted therapies. To meet these needs and recognizing the strong positive link between Multidisciplinary Rounds (MDRs) and improved overall survival, quality of life, and access to clinical trials, the C3 National BTC-MDR have been established [19,20].

The C3 BTC-MDRs occur on a secure virtual Microsoft Teams platform monthly. Following each meeting, the treating oncologist receives a summary report that aids in making treatment decisions. These meetings also allow physicians and trainees to present cases focused on specific knowledge gaps or recent treatment advances. The C3 rounds are accredited by the Royal College of Physicians and Surgeons of Canada, contributing to Continuing Medical Education credits. Each meeting is co-chaired by C3 members and features a dedicated pathologist, radiologist, and molecular oncologist to provide expertise and respond to questions. Though patients cannot attend the rounds, they may request a copy of the summary report of the discussion. Cases can be scheduled through physician requests via an online Case Submission Form or through patient requests via the C3 Navigator. In the inaugural year of the C3 rounds, eight sessions were conducted, reviewing 22 patient cases, with 12 out of 22 cases recommended for a clinical trial or experimental therapy.

By enhancing access to a diverse panel of multidisciplinary expert opinions, BTC MDR aims to provide options and insights into treatment, clinical trials, and targeted therapies. This model of MDR can serve as a reference for other rare cancers, demonstrating the feasibility and benefits of conducting MDR on a national scale.

### 2.3. Molecular Testing and Clinical Trials

Growing research in molecular oncology and immunotherapy has led to new therapies and clinical trials for cholangiocarcinoma, many of which are currently being tested or have received approval. For instance, Health Canada has authorized the FGFR2 (Fibroblast Growth Factor Receptor 2) antagonist pemigatinib for patients harboring an FGFR2 fusion or translocation. Additionally, several clinical trials are investigating molecularly targeted agents that could benefit BTC patients who have actionable mutations, fusions, or amplifications, such as IDH, NTRK, ERBB2, CDK6, and MSI. In molecular oncology, patients with particular mutations may experience nearly twice the median overall survival with targeted therapy, [21]; however, treatment resistance is almost universally encountered. While ongoing research brings hope, accessing these therapies and trials remains difficult.

A major barrier to treating cholangiocarcinoma is due to its rarity. Rare cancers are defined as cancers with an incidence of less than 6 people per 100,000. When examining specific tumor alterations, some BTC subtypes qualify as’ ultra-rare” cancers with an incidence of less than 1 person per 1,000,000 [22], which complicates treatment options and drug accessibility in Canada. This issue stems from an absence of incentives for pharmaceutical companies to allocate their time and resources in Canada, given the extreme rarity of specific BTC subtypes. Consequently, starting a clinical trial poses challenges due to insufficient patient numbers or a lack of compelling evidence concerning specific alterations. To address these challenges, we aim to improve access to therapies, molecular testing, and clinical trials through C3.

Compared to similar-income countries, Canada lags in providing access to molecular testing and innovative clinical trials for BTC. Molecular testing is vital as it reveals important details about a patient’s tumor and can find mutations that might be targeted by specific treatments. It uses advanced sequencing methods and can either be panel-based or cover the whole genome. When the C3 was established, no province or territory funded molecular testing for BTC patients, despite around 45% of BTC tumors having targetable mutations [23,24]. Access and funding for this testing continue to be significant issues for Canadian BTC patients, although Quebec has recently introduced a next-generation sequencing program that is funded by the province, and Ontario and Alberta provide testing for FGFR2 translocations and fusions. While a few research studies and industry-funded programs are available, BTC patients and their oncologists are often unaware of them. For instance, Incyte, the makers of pemigatinib, offered a 170-gene panel through OncoHelix, a commercial molecular lab at the University of Calgary, to help identify patients eligible for pemigatinib due to the presence of the FGFR2 fusion, but the program ended in November 2023.

To facilitate equitable access to molecular profiling for BTC patients across Canada, C3 has created and funded a molecular testing program. Physicians can order molecular testing through the C3 if the patient meets the eligibility requirements and agrees to the Terms and Conditions, which allow C3 to collect and store their medical data within the C3 Registry. Patients in Ontario undergo a custom panel-based molecular test through UHN, in addition to the provincially funded FGFR2 test. Patients in Quebec are directed to the provincially funded Targeted RNAseq 591-gene panel test by OPTILAB, through the McGill University Health Center. All other Canadian patients can receive testing using the 170-gene panel through OncoHelix. The data will increase the feasibility of clinical trials of molecularly targeted therapies in Canada, while also generating RWE of the natural history and access to therapies for these ultra-rare BTC populations. C3 is hopeful that the provision of molecular testing will encourage the industry to open more clinical trials in Canada and more Canadian centers to agree to participate.

### 2.4. Peer-Support Groups and BTC Advocacy

Because BTC is rare, patients and their families have fewer opportunities to connect with others undergoing similar experiences, which can make them feel isolated [16,25]. The C3 monthly support group aims to address this by bringing together individuals affected by BTC, allowing them to share experiences and support one another, regardless of their location in Canada.

The saying “alone we are rare, together we are strong” truly resonates with the BTC community. Advocacy groups for rare cancers face unique challenges—not only in raising awareness and building communities, but also in driving research, establishing patient registries, and advocating for better drug development and access to therapies [26]. In recent years, the crucial role of patient advocacy groups has gained worldwide recognition [27,28]. These are invaluable resources for patients, and clinical teams are increasingly involving patient representatives in steering committees. Patient advocacy groups also play a vital role as partners in rare cancer research consortia [29].

At C3, advocacy means collaborating to demand policy changes and secure increased funding for research and resources. We host a monthly virtual joint advocacy meeting with the Cholangiocarcinoma Foundation and Cholangio-Hepatocellular Carcinoma Canada, which is open to all patients. Tools such as template letters for advocacy are co-developed to help individuals effectively voice their concerns and needs. This approach empowers individuals with rare cancers by providing the resources, support, and platforms they need to amplify their voices and connect with others facing similar challenges.

C3 also participates in global efforts for awareness, such as Cholangiocarcinoma Awareness Month (February) and World Cholangiocarcinoma Day, occurring annually on the third Thursday of every February. Notably, the “Light it Green for CCA” campaign is a powerful advocacy initiative where landmarks across the world light up green to raise awareness. Over 20 sites in Canada were bright green for the 2025 World Cholangiocarcinoma Day, and many of these sites had a local meet-up planned for families and those affected by BTC. Landmarks are illuminated green to honor everyone affected by BTC. Lighting bridges, buildings, and monuments nationwide sparks conversations, promotes early detection, and advocates for better access to diagnostics, treatments, and research funding. This unified effort emphasizes national collaboration in addressing the triumphs and challenges of the BTC community. Additionally, C3 is involved in webinars each year featuring BTC experts and advocates discussing advances in BTC treatment, challenges, and future directions.

## 3. Implementation Measures and Progress to Date

Implementation measures for C3 initiatives focus on ensuring the effective co-design, integration, and delivery of services to patients and healthcare professionals. Maintaining robust and dynamic communication channels plays a key role in driving success. In the field of implementation science, communication and the channels through which it occurs are widely recognized as fundamental to the implementation process [30,31,32,33]. These channels facilitate the exchange of information, align stakeholders, and ensure that evidence-based practices are successfully integrated into ongoing initiatives. C3 integrates strategies from implementation science to optimize communication, enhance collaboration, and promote knowledge sharing. A structured approach is employed for meetings, including biannual steering committee sessions to set strategic directions, quarterly core team meetings to maintain alignment and address emerging issues, and monthly pillar-focused meetings to discuss specific objectives and progress, always with meaningful patient representation and engagement. Through effective communication, the dialog remains collaborative and dynamic, enabling the network to respond to the evolving needs of the BTC community. Key steps include establishing clear protocols for patient enrollment in the C3 National Registry, ensuring equitable access across Canada, and facilitating the collection of both clinical and patient-reported data. To foster continuous improvement, C3 routinely invites and values input from patients and stakeholders. This feedback is carefully considered and used to adjust or enhance program initiatives. Research studies quantitatively measure the impact of C3 initiatives. For example, the “C3 Hope study” measures hope and loneliness levels among C3 patients at baseline, after they meet with the research navigator, and at three months post-engagement with C3. It also aims to identify users’ perceived strengths and gaps in C3 activities. In addition, focus groups provide deeper insights into the experiences of patients and their caregivers. Quantitative data are to be analyzed using repeated measures to capture changes in study variables. To participate in the Hope study, patients and caregivers will sign and provide informed consent. Thematic qualitative analysis will uncover key themes about participants’ experiences. These findings will be reported in distinct manuscripts.

**Figure 2:**
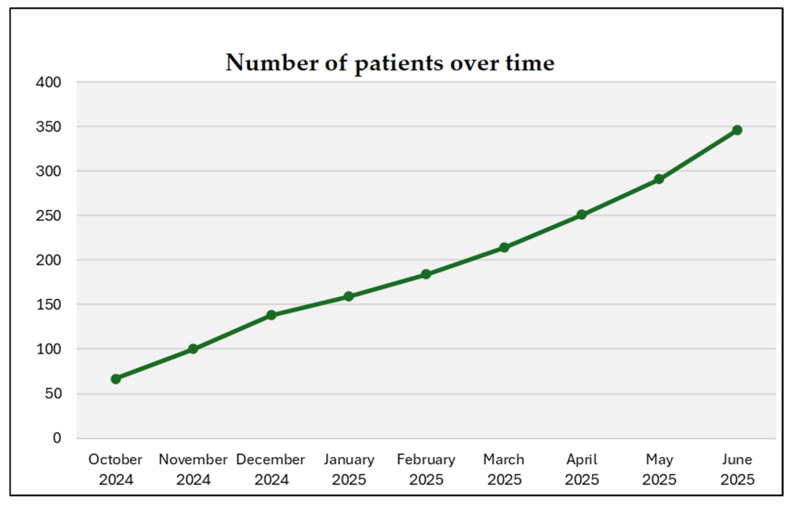
Patients joining the C3 network over time (from October 2024 to June 2025).

C3 also tracks increases in outreach, stakeholder memberships, patient and caregiver enrollment, number of funded molecular testing and trials matching and growing social media presence, reflecting broader community engagement. Together, these activities seek to document implementation processes and provide the foundation for ongoing C3 enhancements. To date (June 2025), approximately 150 healthcare professionals have joined C3, more than 120 molecular tests have been funded, and patients are increasingly hearing about and joining C3. Figure 2 reports the steady increase in C3 patient members until June 2025. Patients are joining C3 from across Canadian provinces (i.e., British Columbia, Alberta, Saskatchewan, Manitoba, Ontario, Quebec, New Brunswick, Nova Scotia, Newfoundland and Labrador).

## 4. Implementation Challenges

C3 initiatives implementation has faced several challenges, including managing health data records, resource constraints, and provincial disparities in healthcare.

Due to its low incidence, research and clinical trials for rare cancers often require pooling participants from different regions to achieve sufficient sample sizes, which is essential for drawing meaningful conclusions and developing effective treatments. However, locating individuals newly diagnosed with BTC nationwide is logistically challenging and resource-intensive.

Another significant challenge is the presence of data silos and fragmented health information systems. This fragmentation hampers the sharing of critical patient information across settings. When health information systems lack interoperability, ensuring continuity of care for patients who move across different healthcare settings or provinces becomes difficult, which is common with rare cancers when treatment or services are located away from patients’ residences. This fragmentation can lead to incomplete patient records, repeated tests, and delays in treatment, all of which can negatively impact patient outcomes.

The limited interoperability between health information systems across provinces exacerbates the problem of fragmented data. Different provinces may utilize various electronic health record systems that are not compatible with one another. Consequently, transferring patient data from one province to another can be cumbersome and inefficient. This lack of seamless data exchange hinders collaborative efforts and makes it challenging to track patient progress and outcomes accurately. Without integrated health information systems such as Citizen Health, C3 goal of providing comprehensive care continuity can be f significantly compromised.

Resource constraints and limited funding are also considered significant challenges for the implementation of C3. Supporting patients and conducting clinical trials require substantial financial and human resources. Unfortunately, resources allocated to rare cancer research and patient support programs are often limited. While the Government of Canada announced an investment of up to $1.5 billion to support the National Strategy for Drugs for Rare Diseases in March 2023, there has not been significant improvement in access to molecular testing or therapies. The limited funding and low prioritization for rare diseases also affect the availability of specialized services, including patient counseling, support groups, and tailored treatment plans.

Canada’s healthcare systems vary significantly across provinces, leading to disparities in healthcare infrastructure and access. These can impact the provision of specialized services, including molecular testing for cholangiocarcinoma, such that even when funding is available, some provinces lack the necessary infrastructure and expertise to conduct advanced molecular testing.

## 5. Conclusions and Future Directions

Cancer navigation programs, whether led by clinicians such as nurses, social workers, or lay navigators, continue to evolve worldwide. Existing navigation programs in North America and Europe encompass information provision, care coordination, psychosocial and financial support, as well as advocacy efforts [3,11,34,35]. C3 is a promising example of an innovative navigation model that connects clinical care with scientific discovery. Moreover, C3 integrates navigation within a research framework, whereby the navigator engages patients and caregivers not just in accessing care but also research while involving relevant stakeholders at every stage—planning, implementation, testing, and beyond. Looking ahead, C3 plans to expand its scope, enhance support networks, explore new care models, and evaluate these through implementation science frameworks. To enhance its national impact, C3 intends to engage in national advocacy efforts across provinces and use the information and resources (patient and clinician networks) to gather key stakeholder feedback on matters relating to care and access to research. This knowledge will boost advocacy efforts at the federal and provincial levels, support nationwide coordination, and promote consistent, equitable access to care across Canada. Through these efforts, C3 will gain valuable insights, especially about early involvement in research, patient experiences, the importance of navigation, and overcoming jurisdictional obstacles—lessons that could benefit other rare disease areas. In summary, the C3 blueprint offers a scalable, adaptable, and transferable model to enhance care and outcomes for people with rare cancers and other conditions.

## Figures and Tables

**Table 1:** C3 initiatives details.

Initiatives	Description
National Patient Registry	Collects real-world data from patients and their caregivers impacted by BTC
National BTC Multidisciplinary Rounds	Provide expert opinions on patient cases and treatment plan. Monthly virtual meetings attended by a multidisciplinary group of specialists
Peer support groups	Offer a dedicated forum for patients and their caregivers to connect with others with similar experiences. Monthly virtual meetings (in French or English)
BTC Advocacy	Engages patients and their families, advocates, clinicians, and researchers through advocacy efforts, providing tools and platforms for policy change and more research funding
Molecular testing	Provides access to molecular testing for eligible patients across Canada through education, navigation, a C3-funded program, and the National Marathon of Hope Whole Genome Transcriptome Sequencing study
Clinical trial matching	Connects patients with clinical trials based on eligibility and medical history

## Abbreviations

The following abbreviations are used in this manuscript:

PCN	Person Centered Navigation
BTC	Biliary Tract Cancer
C3	The Canadian Cholangiocarcinoma Collaborative
MDR	Multidisciplinary Rounds
CCS	Canadian Cancer Society
CIHR	Canadian Institutes of Health Research
TOH	The Ottawa Hospital
REDCap	Research Electronic Data Capture
RWE	Real World Evidence
UHN	University Health Network
TIL	Tumor Infiltrating Lymphocytes
